# Design of Boron Doped C_2_N-C_3_N Coplanar Conjugated Heterostructure for Efficient HER Electrocatalysis

**DOI:** 10.1038/s41598-018-24044-4

**Published:** 2018-04-04

**Authors:** Weiwei Xu, Chongyang Chen, Chao Tang, Youyong Li, Lai Xu

**Affiliations:** Institute of Functional Nano & Soft Materials (FUNSOM), Jiangsu Key Laboratory for Carbon-Based Functional Materials & Devices, Soochow University, 199 Ren’ai Road, Suzhou, Jiangsu 215123 PR China

## Abstract

Hydrogen evolution reaction (HER) via the electrocatalytic reduction of water on metal-free catalysts may become a promising method for a sustainable energy supply in the future. However, compared with noble metals or transition metals, the carbon-based metal-free electrocatalysts show poor activity. Here, a novel coplanar metal-free catalyst (C_2_N-C_3_N) was designed for the first time to achieve better efficiency for electron transfer and water reduction. Through the DFT calculations, we discovered that the unique coplanar C_2_N-C_3_N structure can promote the directional transfer of electrons from C_3_N to C_2_N under the drive of built-in electric potential in the π-conjugated plane. To achieve higher performance in HER, the single atom doping by the substitution of boron is carried out. Remarkably, after the boron is doped, the barrier in the Tafel step decreases from 2.35 eV to 0.86 eV. Our results indicate that the novel B-doped coplanar C_2_N-C_3_N structure is a promising metal-free catalyst for HER.

## Introduction

Nowadays, with the increasingly serious energy crisis and environmental problems, the demand for clean and efficient energy source has become a topic of concern for all. Hydrogen has become one of the most popular resources since it is the cleanest energy carrier. Electrochemical and photoelectrochemical technologies for water splitting are promising ways to store sustainable energy resources (such as solar energy) in the form of hydrogen^1^. Besides, these approaches usually produce energies with high density and do not generate carbon emissions^2^. Therefore, the production of hydrogen energy is of great significance.

In traditional electrocatalytic hydrogen production, noble metals are often used as catalysts, such as platinum and gold^3^. Although noble metals performed well in catalytic hydrogen production, they still have many inevitable shortcomings: (1) difficult to degrade, (2) too expensive^4,5^. Therefore, the study of metal-free catalysts for hydrogen evolution has become a hot topic, and the two-dimensional carbon based materials show remarkable performance in the field of catalysis.

Graphene was discovered as the first two-dimensional material. It has unique electronic, optical and mechanical properties^6^. However, due to its zero band gap, its application in electronic devices is limited. Nitrogen doped materials have similar structures with graphene and have better performance than graphene, such as g-C_3_N_4_ and C_2_N. They were both studied as the photocatalysts for hydrogen evolution by water splitting. Compared with metal-containing materials, they have several advantages^7^: (1) easily to be synthesized; (2) active under visible light illumination. However, they usually have a low activity for catalytic performance. Therefore, strategies for improving their activity have been provided, which include texturization^8–11^, chemical modification^12–14^ and band alignment^15–19^. As a classical modification approach, element doping promotes the charge transfer and the separation of electron-holes, but it also generates new centers for carrier recombination^20,21^.

Recently, designing heterostructures based on 2D materials has become a new advance in the field of catalysis, since it could simultaneously promote charge transfer and suppress the carriers to recombine in bulk materials^22–24^. However, due to the weak interlayer van der Waals force, the generated carriers may still recombine easily in the interlayer space region^25^. To overcome this issue, inducing a built-in electric field by in-plane heterostructure with different work function has become a significant approach^26^. Particularly, 2D carbon-containing materials can be well connected via a π-conjugated structure to form coplanar heterostructures. This structure can improve the transport of electrons, leading to better in-plane electron hole separation and electron transfer, and ultimately enhance the catalytic activity^27,28^. Not long ago, Wei et.al. successfully synthesized the new coplanar heterostructure by covalent bonding of C_3_N_4_ and C-rings. The new structure could quickly trap photoexcited electrons and drive them to suitable active sites, which dramatically enhances the photocarrier separation and catalytic efficiency for HER^29^.

## Results and Discussion

### Construction and stability test of C2N-C3N coplanar structure

**Figure 1:**
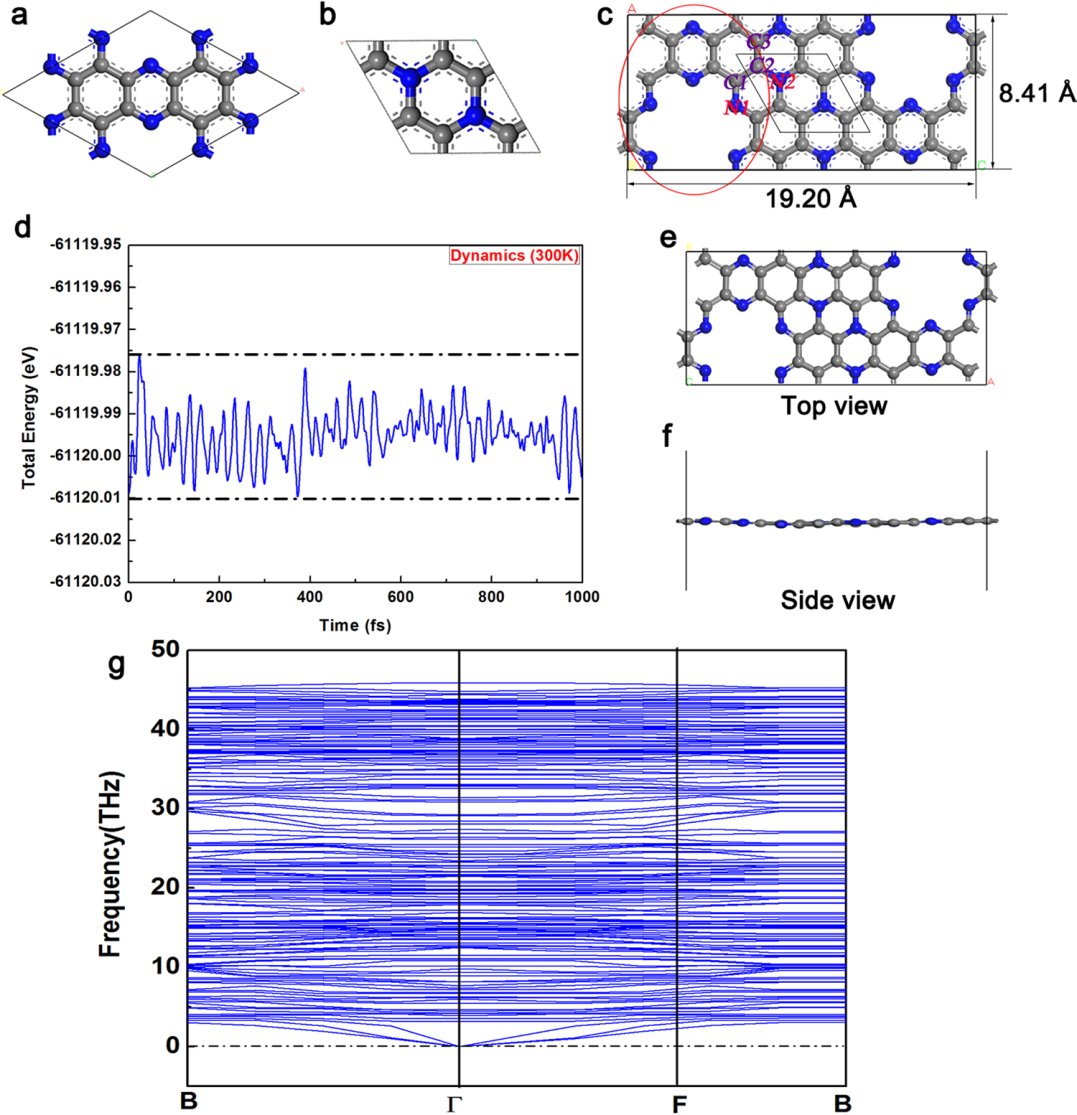
The optimized structure of (**a**) C_2_N, (**b**) C_3_N and (**c**) coplanar C_2_N-C_3_N. The red circle represents the part similar as C_2_N and the parallelogram represents the part of C_3_N. (Gray-carbon atom; blue-nitrogen atom) (**d**) The coplanar C_2_N-C_3_N heterostructure at 300 K during 1 ps from the first-principle molecular dynamics calculations. (**e**) The top-view and (**f**) side -view of the structures after the MD simulations. (**g**) Phonon spectra of C_2_N-C_3_N along the high-symmetric points in the Brillion zone.

Many heterostructures based on C_2_N were studied for water-splitting, because C_2_N has a suitable band position for hydrogen evolution^30^. However, the intrinsic C_2_N structure has a low activity for hydrogen evolution. Herein, to promote charge transfer in two dimensional C_2_N, we designed and predicted a new coplanar heterostructure by connecting C_2_N and C_3_N with π-conjugated bonds for the first time (see Fig. 1c). The lattice parameters of the new structure are as follows: a = 8.41 Å, b = 19.20 Å, c = 20 Å; α = 90°, β = 90°, γ = 90°. The thickness of the vacuum layer in the C_2_N-C_3_N coplanar is 20 Å, which has been proved to be stable by the tests (shown in Figure S1). In order to illustrate the stability of the novel structure, the molecular dynamics calculations were performed for C_2_N-C_3_N coplanar structure (see Fig. 1d–f). It was found that the maximum fluctuation of the total potential energy is smaller than 0.04 eV/unit cell through MD calculations and the structure has no obvious breakage at 300 K. Furthermore, the dynamical stability of C_2_N-C_3_N coplanar heterostructure is tested by calculating the phonon spectra, as shown in Fig. 1g. It can be clearly seen that no imaginary frequency phonons are found at any wave vector, which demonstrates that C_2_N-C_3_N coplanar structure is dynamically stable. Compared with graphene@g-C_3_N_4_ coplanar heterostructure, C_2_N-C_3_N has more similar parts in terms of structures and element components between C_2_N and C_3_N, and the nitrogen atoms with rich long-pair electrons at the edge of the conjugated rings provide more ideal sites for interfacial connection of heterostructures^31^. Through the calculations, our results have demonstrated that the novel coplanar C_2_N-C_3_N significantly promotes the electrons transfer through the π-conjugated structure. Moreover, the B-doped C_2_N-C_3_N coplanar structure effectively reduces the activation energy in Tafel step, indicating a good performance for HER.

### Electronic properties of C2N-C3N coplanar structure

**Figure 2:**
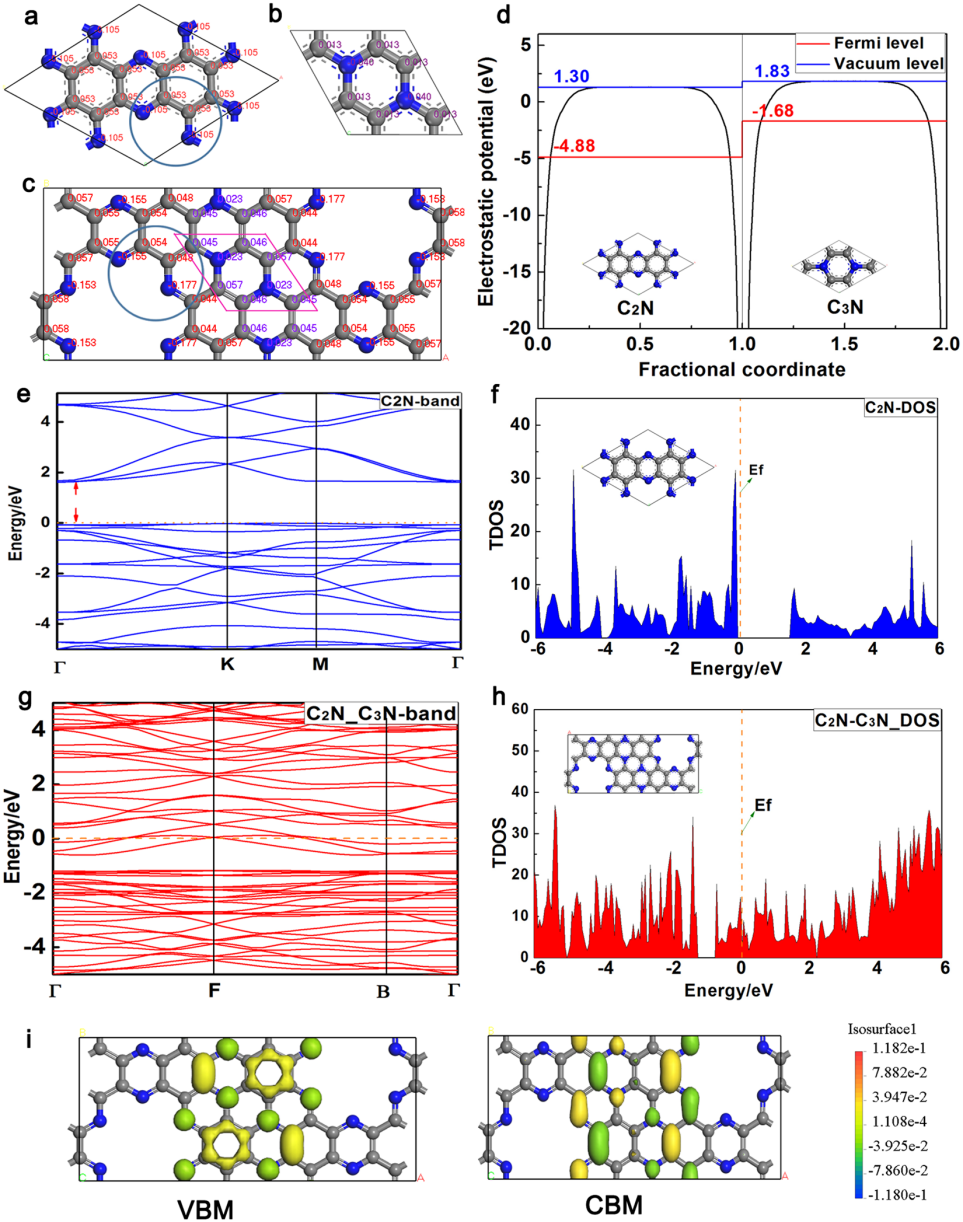
The charge distribution on (**a**) C_2_N, (**b**) C_3_N and (**c**) C_2_N-C_3_N, respectively. (**d**) The electrostatic potential of C_2_N and C_3_N, (the blue line represents the vacuum level and the red line represents the Fermi level). The band structures of (**e**) pure C_2_N and (**g**) coplanar C_2_N-C_3_N. The density of states on (**f**) pure C_2_N and (**h**) coplanar C_2_N-C_3_N. (**i**) The charge distribution of the VB and CB band edges with the isosurface label.

It’s known that the process of charge transfer plays an important role in catalytic reactions. To compare the charge distribution of C_2_N (C_3_N) with the new coplanar heterostructure of C_2_N-C_3_N, the population analysis was performed by assigning Hirshfeld charge on them. As shown in Fig. 2a–c, after the heterostructure is formed, the charge of the most atoms in coplanar heterostructure (marked by blue circle) exhibits a more negative charge (0.105/−0.155, 0.053/0.018, 0.105/−0.177) than C_2_N. Similarly, compared with C_3_N, the charge in the same carbon atoms in the new structure shows a more positive charge (0.013/0.045, 0.013/0.046, 0.013/0.057), but the nitrogen atoms had more negative charge. This phenomena shows that the overall negative charge is transmitted from C_3_N to C_2_N in the new coplanar C_2_N-C_3_N structure, and nitrogen atoms achieve more negative charge in the C_3_N itself. That is to say, the electrons shifted directionally from C_3_N to C_2_N in the new coplanar structure and it would have a significant impact on catalytic performance.

To give a sound explanation of the directional migration of electrons, the electrostatic potentials of C_2_N and C_3_N were calculated in order to compute the work function. Work function is the potential required to remove the least lightly bound electrons: (*ɸ*_w_: work function, E_V_: the energy of vacuum level, E_f_: the energy of Fermi level), which refers to the minimum energy that an electron escapes from the Fermi energy level into vacuum energy level^32^. As illustrated in Fig. 2d, the work function of C_2_N and C_3_N equal 6.18 eV and 3.51 eV respectively. Consequently, the difference of *ɸ*_w_ between C_2_N and C_3_N results in a strong built-in electric field, which is the main reason for the directional migration of electrons. In the heterostructure, the electrons would transfer from one side with the low work function to the higher one. The *ɸ*_w_ of C_2_N is larger than that of C_3_N, so when the connection takes place, the internal electrons would transfer from C_3_N to C_2_N directionally, which would increase conductivity and would promote catalytic activity.

For an in-depth understanding of the difference of the electronic structures between the pristine C_2_N and coplanar C_2_N-C_3_N, the band structure and total density of states (TDOS) were obtained by PBE calculation. As seen from Fig. 2e, the pristine C_2_N presents a typical characteristic of semiconductor, with a band gap of 1.68 eV, which is consistent with the previous work^33^. However, the coplanar C_2_N-C_3_N hybrid displays a feature of conductor with no band (Fig. 2g). In Fig. 2f, the Fermi level is located in the middle of the valance band (VB) and conduction band (CB) without any electron distribution there, and has a gap of 1.68 eV. However, in Fig. 2h the Fermi level crosses the conduction band of the coplanar of C_2_N-C_3_N. This change in total density of states between the pristine C_2_N and C_2_N-C_3_N demonstrates the electron mobility achieves great enhancement in the coplanar C_2_N-C_3_N structure, which has an important effect on the electrocatalytic HER. These properties are consistent with the previous discussion about charge transfer (in Fig. 2a–c). Furthermore, the charge distribution of the valance band maximum (VBM) and conduction band minimum (CBM) were calculated. When the C_2_N-C_3_N heterostructure was excited, the electrons would be excited from VBM to CBM. As seen from Fig. 2i, the electrons would transfer from the middle of C_3_N to the adjacent edge of C_2_N, which also confirms the existence of the built-in electric field.

### Adsorption of H and H2 on C2N-C3N coplanar structure

As the initial step for both dissociative and associative mechanism, the adsorption of H is significant in the whole process in HER. If the H is weakly adsorbed on the surface, the adsorption step would limit the overall reaction rate. If the adsorption is too strong, the reaction of desorption will limit the reaction rate. Therefore, we first calculated the adsorption energy of H on the coplanar C_2_N-C_3_N. Several possible adsorption sites were considered, including C1, C2, C3, N1 and N2 atoms (see Fig. 1c). As shown in Table 1, we can see that the adsorption of H on these selective sites all belong to chemisorption according to the adsorption energy analysis and the bond length analysis. It can be found that N1 atom has the strongest adsorption energy (−3.47 eV), which indicates that it could adsorb H easily but it may be too hard to be released. Moreover, the N2 site has the smallest adsorption energy (−1.53 eV), so it may not be beneficial for the H adsorption. However, the E_ads_ of the three carbon sites are −2.22 eV, −2.33 eV and −2.33 eV respectively, and the distance between H and C are all about 1.11 Å. These adsorption on carbon sites (C1, C2, C3) all indicate chemisorption and the adsorption energy is more suitable for the adsorption and release of H than that on the nitrogen sites (N1, N2). On the other hand, as the last step of hydrogen evolution in HER, the adsorption of H_2_ molecule is also very important. It can be seen from Table 1 that the adsorption energy on these selective sites are all about −0.20 eV and the length between H and N(C) are all more than 2.65 Å. This result indicates that the adsorption of H_2_ on the coplanar C_2_N-C_3_N is the physisorption, which facilitates the release of hydrogen. In combination with the adsorption of H and H_2_, the carbon sites may be more favorable to be active sites for HER reaction. Moreover, the C1 is located at the edge of the hole which is easier to be exposed outside, so C1 is considered as the most favorable active site for the HER reaction to take place.

**Table 1:** Adsorption energy (E_ads_, eV) for HER Intermediates and bond length (Å) on C_2_N-C_3_N coplanar structure.

		N1	N2	C1	C2	C3
H_ads_/eV	E_ads_	−3.47	−1.53	−2.22	−2.33	−2.33
	Length	1.024	1.037	1.113	1.109	1.109
H_2ads_/eV	E_ads_	−0.21	−0.19	−0.17	−1.08	−0.18
	Length	2.686	2.774	2.829	2.897	2.885

### HER pathways on C_2_N-C_3_N coplanar structure

The HER mechanism is generally considered to have three possible reaction steps (see Table 2)^34^. In both cases, HER takes two protons into hydrogen molecule:

1 $${{\rm{2H}}}_{3}{{\rm{O}}}^{+}+2{e}^{-}\to {{\rm{H}}}_{2}\uparrow +{{\rm{2H}}}_{2}{\rm{O}}.$$

**Table 2:** The reaction steps of two kinds of mechanisms of HER.

	Reaction Steps
Mechanism 1	Volmer ---- ${{\rm{H}}}_{3}{{\rm{O}}}^{+}+{e}^{-}\to {{\rm{H}}}^{\ast }+{{\rm{H}}}_{2}{\rm{O}}$
	Heyrovsky ---- ${{\rm{H}}}^{\ast }+{{\rm{H}}}_{3}{{\rm{O}}}^{+}+{e}^{-}\to {{\rm{H}}}_{2}\uparrow +{{\rm{H}}}_{2}{\rm{O}}$
Mechanism 2	Volmer 1---- ${{\rm{H}}}_{3}{{\rm{O}}}^{+}+{e}^{-}\to {{\rm{H}}}^{\ast }+{{\rm{H}}}_{2}{\rm{O}}$
	Volmer 2 ---- ${{\rm{H}}}^{\ast }+{{\rm{H}}}_{3}{{\rm{O}}}^{+}+{e}^{-}\to 2{{\rm{H}}}^{\ast }+{{\rm{H}}}_{2}{\rm{O}}$
	Tafel ---- ${{\rm{2H}}}^{\ast }\to {{\rm{H}}}_{2}\uparrow$

Herein, we took a series of configuration optimization and transition state search for the calculation of activation energy for HER on the coplanar C_2_N-C_3_N. According to the analysis of adsorption energy on the selective active sites, we chose the C1 as the active site for HER.

**Figure 3:**
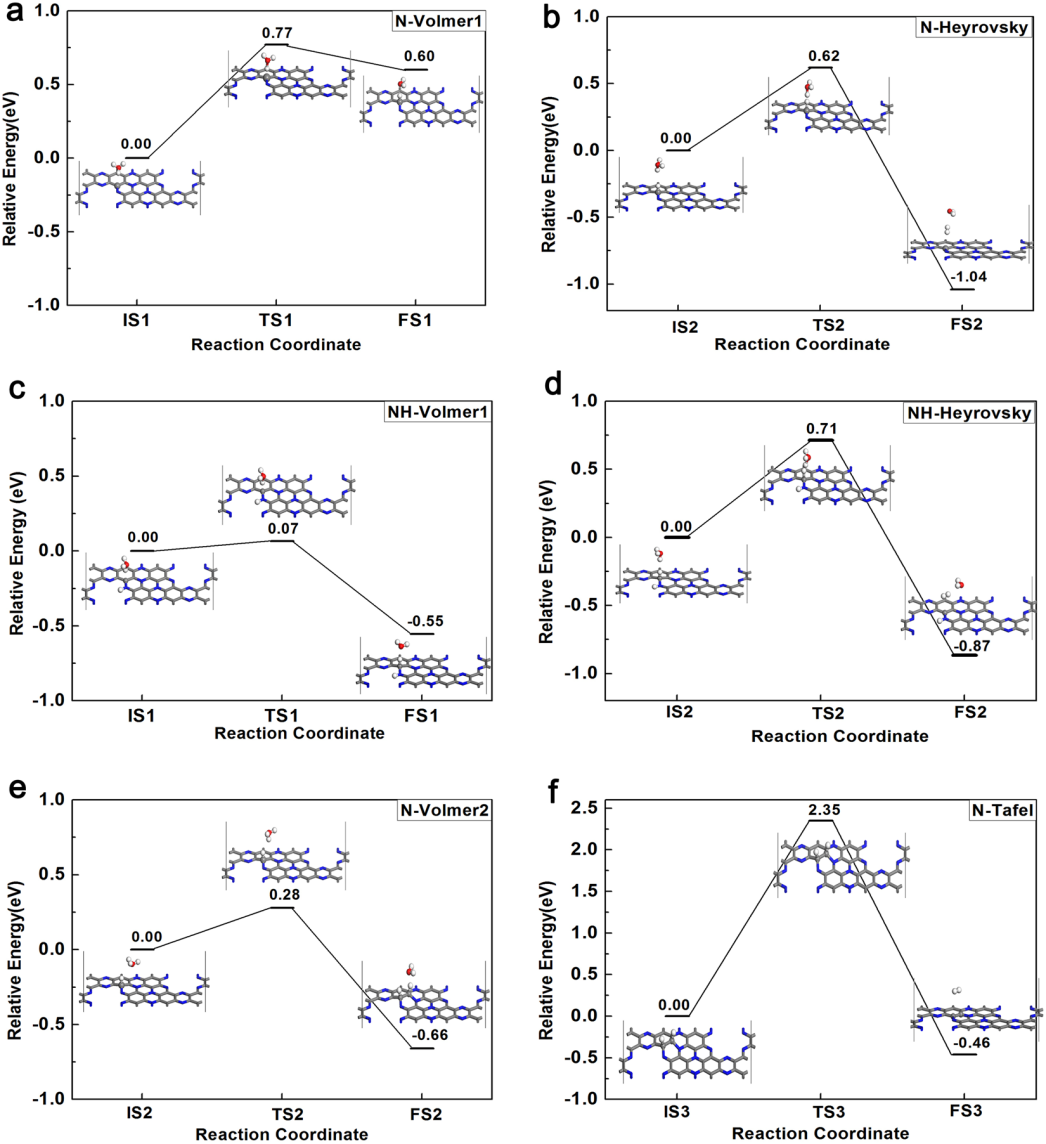
Optimized structures of initial (IS), transition (TS) and final (FS) states of Volmer- Heyrovsky steps and the corresponding activation barrier for each step on coplanar C_2_N-C_3_N (**a**,**b**) and coplanar C_2_N-C_3_N with the N1 adsorbed with H (**c**,**d**). Optimized structures of IS, TS and FS of Volmer (**e**)-Tafel (**f**) steps on coplanar C_2_N-C_3_N, and the corresponding activation barrier for each step.

Hence, we firstly simulated the Volmer-Heyrovsky mechanism on the C1 sites. The activation barrier and the structures of IS, TS, FS (initial state, transition state and final state) are depicted in Fig. 3a,b. At the transition state in the Heyrovsky, the adsorbed H is beginning to break away from C1 and is close to the other H atom. Across the TS, the final state with a weak adsorption of H_2_ molecule above the surface was formed with the H-H bond length being 0.75 Å. For the Volmer reaction, the activation energy is 0.77 eV, which is relatively high in the first step of H adsorption. This phenomenon may be due to the fact that the first H is more beneficial to be adsorbed on the N1 atom. However, based on the discussion on the adsorption on the N1 atom, it’s hard for H to release from N1. Also, previous work has confirmed that the H coverage on sulfur atom of MoS_2_ has a great effect on the adsorption of H and the activation barrier of HER^35,36^. Hence, we performed the same simulation under the condition with H adsorbed on N1. In Fig. 3c,d, as we expected, the activation energy has a marked decline, which changes from 0.77 eV to 0.07 eV. And the energy of the product in the Volmer reaction becomes lower than that of the reactants. These results show a more reasonable reaction path when the H atom covers on the N1 atom. However, in the Heyrovsky reaction, the activation barrier of the H coverage (0.71 eV) is a little larger than that without H coverage (0.62 eV). This is because the FS in the Volmer of the structure with H adsorbed in N1 has much lower energy than that without H coverage. In general, the adsorption of H on N1 has a positive effect on the reaction path of the mechanism of Volmer-Heyrovsky.

We next turned to the second mechanism (Volmer-Tafel), where the two H adsorbed on the C1 and C2 sites react to form H_2_. In this mechanism, the first H adsorbed on the C1 is the same as the Volmer-Heyrovsky, so we just simulated the step of the second H adsorbing on C2 sites and the step of H_2_ formation. In the Volmer2 step, the activation energy is about 0.28 eV, with favorable reaction energy of −0.66 eV (see Fig. 3e). The last step is Tafel reaction (see Fig. 3f). In the IS, the distance between the two H (bonded to C1 and C2) is 2.12 Å. Then they approach and form the transition state with the distance of 1.35 Å. In the final state, the evolved H_2_ released from the surface of the coplanar with the H-H bond length of 0.75 Å. The reaction energy is about −0.46 eV, which is relatively favorable. However, the activation energy is 2.35 eV and it may not be easy for the reaction to take place at room temperature. Similarly, considering the effect of H coverage, we also simulated the process of Tafel reaction when H adsorbs on N1 (see Figure S2). The activation energy doesn’t show the same decrease which further confirms that the Tafel route is less favorable than the Heyrovsky route for this coplanar structure. The reason for this phenomenon may be that the reaction sites (C1 and C2) still don’t have a sufficient activity for HER. As reported by Jiao *et al*. the carbon-based metal-free electrocatalysts generally demonstrate poor activity and the heteroatom-doped methods were studied for a better performance for HER^37^. Furthermore, the boron doped graphene has been reported with efficient electrocatalytic activity for HER^38^.

### HER pathways on B-doped C_2_N-C_3_N coplanar structure

**Figure 4:**
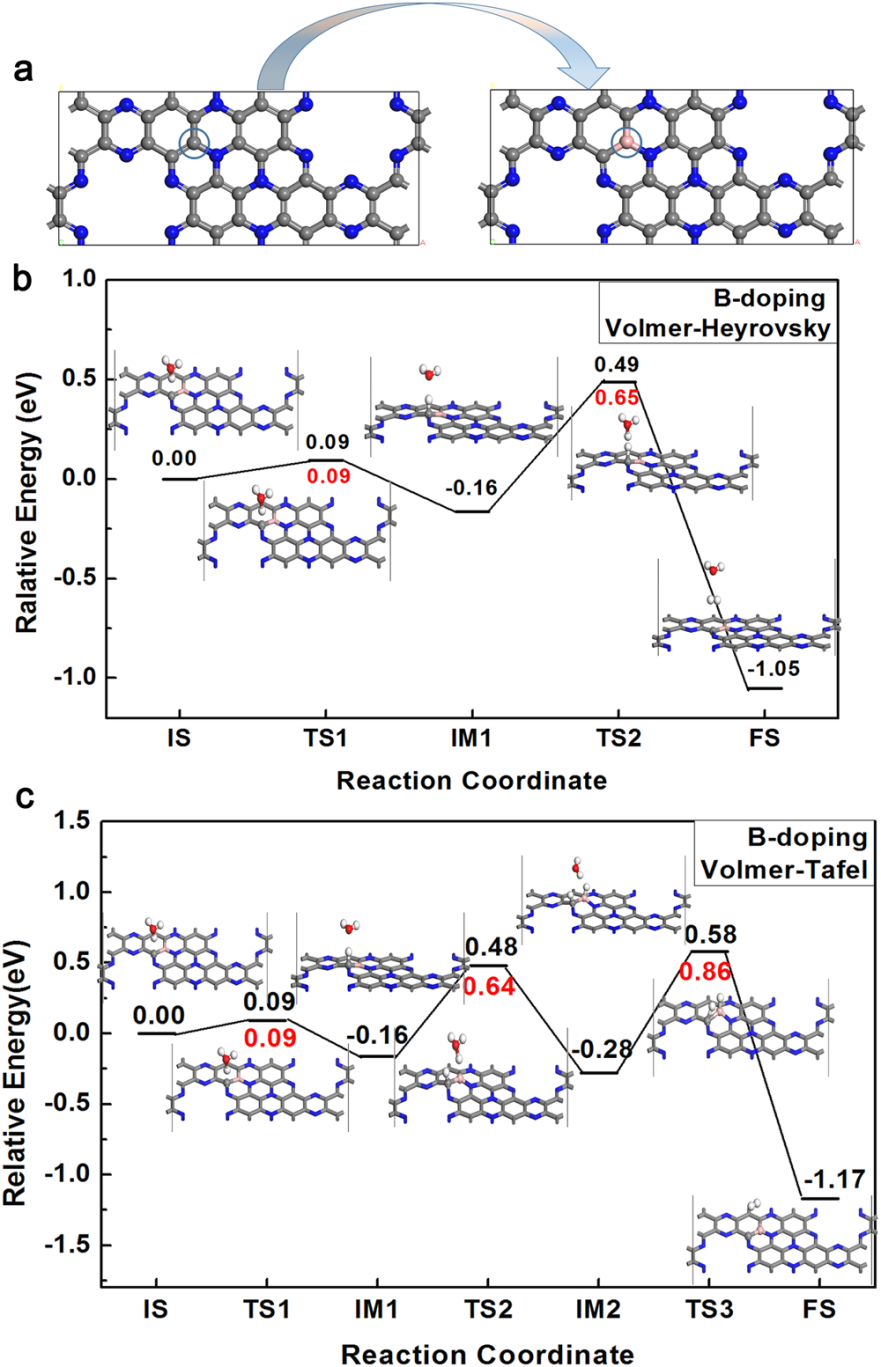
The top view of the pristine coplanar C_2_N-C_3_N and the coplanar with the B doped on C_2_ site (**a**). The circled atom is the C_2_ atom and B atom respectively. Optimized structures of IS, TS and FS of Volmer-Heyrovsky mechanisms (**c**) and Volmer-Tafel (**d**) mechanisms on B-doped coplanar C_2_N-C_3_N, and the corresponding activation barrier for each step.

In order to confirm our guess and improve the catalytic performance of Tafel mechanism in HER on the coplanar C_2_N-C_3_N, the C2 atom was doped by boron atom as the active sites for H_2_ evolution (see Fig. 4a). The doping concentration of B is 2.95% (the ratio of the number of doped atoms to the substituted atoms when they are not doped). We next calculated the activation barrier in both Volmer-Heyrovsky and Volmer-Tafel mechanisms. As depicted in Fig. 4b, it shows the pathways of the Volmer-Heyrovsky mechanisms with the activation energy and the structure of IS, TS and FS. In the Volmer step, the activation barrier is 0.09 eV, and the reaction energy is −0.16 eV. In Heyrovsky step, the activation barrier is 0.65 eV and the reaction energy is −0.89 eV. It also shows that the Heyrovsky mechanism is the dominant step and all the energy change in the reactions is acceptable with the B-doped C_2_N-C_3_N structure. On the other hand, as shown in Fig. 4c, it shows the overall reaction pathway of Volmer-Tafel mechanism. Similarly, the first step in Volmer-Tafel is the same as that in Volmer-Heyrovsky. In the Volmer2 step, we found that the activation energy is 0.64 eV, which is 0.36 eV larger than the case without doping. However, in the Tafel step, the barrier is 0.87 eV, which has a 1.48 eV decrease than the case without doping, and makes the Volmer-Tafel mechanism possible to occur. By the calculation of the adsorption energy of H on the B atom, it was found that the E_ads_ of H on B atom is 1.82 eV and is 0.51 eV lower than that on C2. Thus, the doping of B atom shows a lower activity on the adsorption on H in the Volmer2, while it also shows a better performance on the release of H_2_ in the Tafel reaction. Therefore, we predicted that the HER catalytic activity of the coplanar C_2_N-C_3_N can be improved by doping B atoms, with the Volmer-Heyrovsky and Volmer-Tafel mechanism being both favorable at room temperature.

## Conclusion

In summary, we have designed a novel coplanar heterostructure by connecting C_2_N and C_3_N to achieve efficient electron transfer, which facilitates the electrocatalytic reduction of water to H_2_. From the analysis of electronic properties, we discovered that the electrons have a directional transfer from C_3_N to C_2_N under the built-in potential caused by the work function difference. Also, the electron density distribution located at Fermi level indicates that the novel structure possesses good conductivity and accelerates the charge transfer. Based on the TS search of the whole pathways on the pristine C_2_N-C_3_N for HER, it was found that the barrier of the Tafel step is 2.35 eV, which cannot take place at room temperature. However, by the doping of single boron atom, there is a significant decrease of activation energy of the Tafel step from 2.35 eV to 0.86 eV, which makes both HER mechanisms (Volmer-Heyrovsky and Volmer-Tafel) become favorable at room temperature. Our results indicate that the novel B-doped C_2_N-C_3_N coplanar heterostructure is a promising electrocatalyst for HER and also provides opportunities for the future design of metal-free, low-cost and high-efficiency catalysts for the production of clean energy.

## Methods

Most of the simulations were based on density functional theory (DFT)^39^ and carried out in Dmol^3^ program^40^. The generalized gradient approximation with Perdew-Burke-Ernzerh functional was chosen to describe the electronic interaction effects^41,42^. The basis set of DNP and the basis file of 3.5 was chosen in electronic column, when the involved structures were optimized. The SCF was set with a convergence value of 1.0 × 10^−5^ Ha to the orbital occupation, which was employed to enhance SCF convergence efficiency^43–45^. The cut-off energy was set to be 500 eV, and the K-points was set as 4 × 5 × 1.

To calculate the adsorption energy of small molecules on the substrate, we provide a definition for the calculation of the adsorption energy (E_ad_) as:

2 $${E}_{ad}={E}_{sub\& med}-({E}_{med}+{E}_{sub})$$

where E_sub&med_, E_med_ and E_sub_ are the total energy of the substrate with the adsorbed mediate molecule, a single mediate molecule and the substrate respectively. The transition state search is carried out in the DMol^3^ module, with the method of the complete linear synchronous transit/quadratic synchronous transit (LST/QST)^46^. The energy barrier (E_b_) of the reactions is defined as:

3 $${E}_{b}={E}_{TS}-{E}_{IS}$$

where E_TS_ and E_IS_ are the energy of the transition state and the initial state. Furthermore, first-principle molecular dynamics calculations (MD) are also performed to estimate the structural thermal stability, and the temperature was set at 300 K with the canonical ensemble (NVT) used^47^. The phonon spectra was calculated to test the dynamical stability of the structure by CASTEP^48^. The VBM and CBM of the heterostructure was calculated to investigate the transfer of electrons when the structure was excited.

Electronic properties calculations (dos and band structures) were implemented in the Vienna Ab initio Simulation Package (VASP)^49^. The generalized gradient approximation (GGA) is realized by Perdew-Burke-Ernzerhof (PBE) functional with projected augmented wave (PAW) method. The cut-off energy was set to be 500 eV, which is accurate enough to describe the outer valence electrons at p orbital of B, C and N atoms. The convergence criteria of energy and force are 1.0 × 10^−6^ eV per atom and 0.02 eV/Å, respectively. The GGA-PBE functional with 4 × 5 × 1 K-points for the unit cell was used to characterize the electronic properties and band structures.

## Electronic supplementary material

Supplementary Information
