# Supplementary material for: Design of Boron Doped C2N-C3N Coplanar Conjugated Heterostructure for Efficient HER Electrocatalysis
Source: Sci Rep. 2018 Apr 4;8:5661. doi: 10.1038/s41598-018-24044-4 (PMC5884811; doi:10.1038/s41598-018-24044-4)
Supplement: Supplementary file 1 — Supplementary Information [file 41598_2018_24044_MOESM1_ESM.pdf]

Supplementary Information

Design of Boron Doped C_2N-C_3N Coplanar Conjugated Heterostructure for Efficient HER Electrocatalysis

Weiwei Xu, Chongyang Chen, Chao Tang,Youyong Li, Lai Xu*
Institute of Functional Nano & Soft Materials (FUNSOM), Jiangsu Key Laboratory for Carbon-Based Functional Materials & Devices, Soochow University, 199 Ren'ai Road, Suzhou, Jiangsu 215123, PR China

[*] Email:**xulai15@suda.edu.cn**

The thickness of the vacuum layer in the C_2N-C_3N coplannar structure is 20 Å, which is proved to be stable by the tests (shown in **Figure S1**).

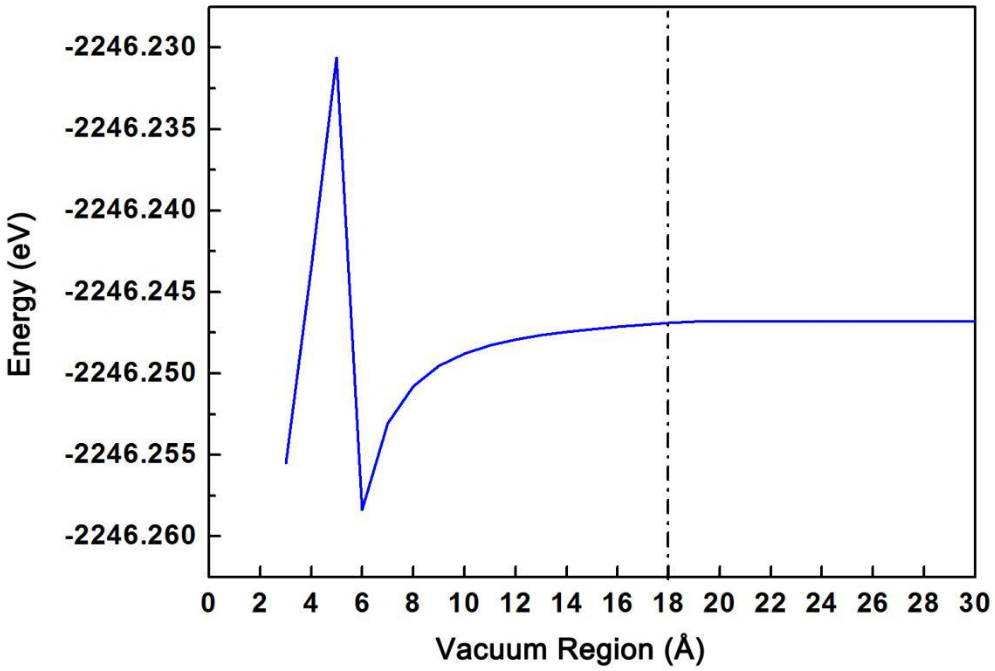

Figure S1. The energy fluctuate of the heterostructure was tested with the change of the thickness of the vacuum layer from 3 Å to 30 Å.

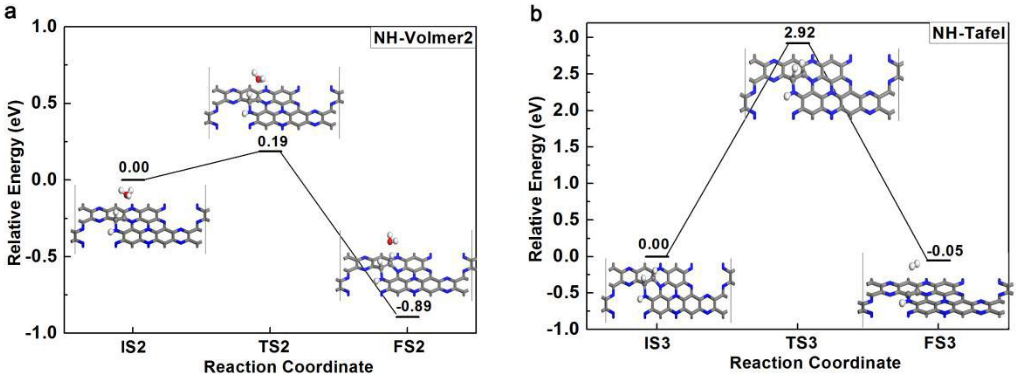

Figure S2. Optimized structures of IS, TS and FS of Volmer(a)-Tafel(b) steps on coplanar C_2N-C_3N with the N1 adsorbed with H, and the corresponding activated barrier for each step.
